# Impact of Measurement Uncertainties on Receptor Modeling of Speciated Atmospheric Mercury

**DOI:** 10.1038/srep20676

**Published:** 2016-02-09

**Authors:** I. Cheng, L. Zhang, X. Xu

**Affiliations:** 1 Air Quality Research Division, Science and Technology Branch, Environment Canada, 4905 Dufferin Street, Toronto, Ontario, M3H 5T4, Canada; 2 Department of Civil and Environmental Engineering, University of Windsor, 401 Sunset Avenue, Windsor, Ontario, N9B 3P4, Canada

## Abstract

Gaseous oxidized mercury (GOM) and particle-bound mercury (PBM) measurement uncertainties could potentially affect the analysis and modeling of atmospheric mercury. This study investigated the impact of GOM measurement uncertainties on Principal Components Analysis (PCA), Absolute Principal Component Scores (APCS), and Concentration-Weighted Trajectory (CWT) receptor modeling results. The atmospheric mercury data input into these receptor models were modified by combining GOM and PBM into a single reactive mercury (RM) parameter and excluding low GOM measurements to improve the data quality. PCA and APCS results derived from RM or excluding low GOM measurements were similar to those in previous studies, except for a non-unique component and an additional component extracted from the RM dataset. The percent variance explained by the major components from a previous study differed slightly compared to RM and excluding low GOM measurements. CWT results were more sensitive to the input of RM than GOM excluding low measurements. Larger discrepancies were found between RM and GOM source regions than those between RM and PBM. Depending on the season, CWT source regions of RM differed by 40–61% compared to GOM from a previous study. No improvement in correlations between CWT results and anthropogenic mercury emissions were found.

Receptor models have been used in the source apportionment of atmospheric mercury (Hg) and other air pollutants. These models make use of ambient monitoring data as well as back trajectory data in some models for the purpose of identifying potential types of emission sources, atmospheric Hg processes, and locations of major source regions. A summary of the major receptor methodologies and findings from speciated atmospheric Hg studies have been published in a review paper^1^. One of the recommendations from the review paper is assessing the extent that uncertainties in gaseous oxidized Hg (GOM) and particle-bound Hg (PBM) measurements affect receptor modeling results.

GOM and PBM are operationally-defined Hg(II) compounds, such as mercuric halides, mercuric sulfate, mercuric nitrite, and mercuric hydroxide^2^. Measurement of GOM and PBM in ambient air is challenging because they are typically present in trace concentrations^3^ (pg m^−3^ range). The exact chemical forms have not been determined; therefore, calibration methods are not available to determine the accuracy and uncertainties of GOM and PBM measurements. Currently the uncertainties are estimated from intercomparison of measurements from different Hg measurement methods. Other sources of measurement uncertainties during sampling include ozone and water vapor interferences^4^,^5^,^6^, chemical reactions^2^,^7^,^8^, and variable collection efficiencies depending on the form of GOM^9^,^10^. These uncertainties are believed to be contributing to the underestimation of GOM. PBM measurements may be biased low or high and the extent of the uncertainties are not well-established^3^. Furthermore, GOM may not be easily separated from PBM during sampling and analysis due to the temperature dependency of Hg(II) gas-particle partitioning. Therefore, it may be considered more accurate to combine GOM and PBM into a single reactive Hg parameter^3^,^11^.

In this study, the effect of GOM measurement uncertainties on Principal Components Analysis (PCA), Absolute Principal Component Scores (APCS), and Concentration-Weighted Trajectory (CWT) receptor modeling results were investigated. The study utilized the same receptor methodologies as in previous studies^12^,^13^; however, the atmospheric Hg data have been modified in two ways to improve the data quality. The first approach combines GOM and PBM into a single reactive Hg compound, instead of separating GOM and PBM as in previous studies. The second approach excluded low GOM measurements, which is a method of data quality control to handle below detection limit (DL) data. Comparison of the receptor model results from this study to previous studies may determine which approach would be most effective in reducing the receptor model uncertainties and could be an alternative method for analyzing atmospheric Hg data until calibration methods become available. Results from this study can also provide an uncertainty assessment of the previously-obtained atmospheric Hg receptor modeling results.

## Methods

### Receptor models

PCA, APCS, and CWT models were employed in this study, identical to the receptor models in Cheng *et al.*^12^,^13^. PCA has been applied to a set of correlated air pollutants and meteorological data to infer potential emission sources and atmospheric processes affecting receptor measurements. PCA component loadings indicate the degree of association between the pollutant or meteorological parameter and a component. Parameters with high component loadings are used to infer potential sources and processes. APCS is derived from PCA and indicates the extent that each of the sources or processes affects each receptor measurement. CWT is a trajectory residence time model that is weighted by the atmospheric Hg concentration corresponding to the arrival of each trajectory. Therefore, it shows the spatial distribution of potential source locations, defined by CWT values ≥90^th^ percentile, contributing to the receptor measurements. Refer to Cheng *et al.*^12^,^13^ for detailed PCA, APCS and CWT procedures undertaken in this study.

### Datasets

For PCA and APCS, the dataset consisted of daily average concentrations of atmospheric Hg, ancillary pollutants, and meteorological data measured between January 2009 and December 2010 from the Kejimkujik National Park (KEJ) site. This is a coastal rural site in Nova Scotia, Canada ~60 km from the Atlantic Ocean. Three forms of atmospheric Hg were measured using a Tekran Hg speciation system: gaseous elemental Hg (GEM), GOM, and PBM <2.5 μm. In the first two hours of sampling, GEM was measured every 5 min while GOM and PBM were collected. In the third hour, GOM and PBM were desorbed and quantified. The elevation of the Tekran instruments was 170 m above sea level and the sample inlet was 5 m above ground. Ancillary pollutants obtained from CAPMoN^14^ include trace gases (SO_2_ and HNO_3_), particulate inorganic ions (Ca^2+^, K^+^, Mg^2+^, Na^+^, Cl^−^, NH_4_^+^, NO_3_^−^, and SO_4_^2−^) and ground-level O_3_. PM_2.5_ concentrations were obtained from the National Air Pollution Surveillance Network^15^. Meteorological data from the Historical Climate Data website^16^ were used in PCA and included temperature, relative humidity, wind speed, and precipitation. Further details on the KEJ dataset can be found in Cheng *et al.*^12^.

For the CWT model, the dataset consisted of 3-hr GEM, GOM and PBM concentrations measured between January 2010 and December 2011 from downtown Dartmouth, Nova Scotia, Canada. The Dartmouth site is located in an urban area that is part of the Halifax Regional Municipality and is ~15 km from the Atlantic Ocean. Hg concentrations were measured using a Tekran speciation system situated on the roof of a three-story building. The instruments also operated on 3-hr sampling/desorption cycles similar to the instrument at the KEJ site. Trajectory segment endpoints data corresponding to each 3-hr Hg measurement were computed using the HYSPLIT back trajectory model^17^,^18^. The number of trajectory endpoints over each grid cell was determined and then used in conjunction with Hg concentrations to calculate the CWT for each grid cell as described in Cheng *et al.*^13^.

### Treatment of GOM and PBM data

In previous studies^12^,^13^, GEM, GOM and PBM concentrations without any data manipulation were used in the receptor models, referred to as SpecHg, GOM, and PBM reference cases in Table 1. In the SpecHg case, all 3-hr Hg concentrations including low GOM measurements were used to calculate the daily averages that were then inputted into the PCA model. Daily averages were calculated for the Hg data because daily trace gas and particulate ion measurements were also included in the dataset analyzed by PCA. PCA was based on pairwise analysis of correlation coefficients for each pair of parameters with available daily averages, instead of listwise analysis which performs the correlation only when the daily average for all parameters is available. The pairwise option uses more of the data, which is advantageous when there are missing daily averages in any parameter due to instrumentation issues or insufficient number of 3-hr measurements to compute a valid daily average. The GOM and PBM reference cases used in the CWT model included all 3-hr concentrations.

In this study, the same receptor models were used with different sets of Hg data, and the results were compared with the reference cases. One dataset summed GOM and PBM concentrations into a single parameter defined as reactive Hg (RM cases, Table 1). RM was calculated only when both GOM and PBM data were available; otherwise, it was treated as missing. The second datasets were subjected to further data quality control (qc) by removing low GOM concentrations defined by percentiles of a probability distribution. There are various methods to treat low measurements (e.g. <DL) of environmental data prior to applying multivariate analysis, such as substitution with zero, DL, DL/2 or random values between zero and DL, or retaining low measurements. The treatment of low measurements depend on the total number of measurements, number of measurements <DL, and the probability distribution of the measurements^19^,^20^. Given the challenges of quantifying trace amounts of GOM in air and the biased low measurements of GOM, removing low GOM concentrations could reduce the uncertainties in the data. Instead of selecting invariable cut-off concentrations, percentiles were used as the cut-off concentrations because of the pronounced log-normal distribution of the GOM data. In previous studies, percentile cut-offs were also used to evaluate the effects of different data treatments for <DL data on multivariate analysis results^19^,^20^. For PCA, GOM concentrations ≤10^th^ and ≤50^th^ percentiles for the entire 2009–2010 period were excluded from the dataset prior to calculating the daily average (GOMqc-10 and GOMqc-50 cases, respectively, in Table 1). The 10^th^ and 50^th^ percentile GOM concentrations at the KEJ site were 0 and 0.24 pg m^−3^, respectively. Even at 50^th^ percentile, the GOM concentration was very low at the KEJ site. Selecting a lower percentile as the cut-off concentration may have insignificant impact on the receptor model results. Daily average GOM was calculated only when there was a minimum of four 3-hr measurements per day. For CWT, 3-hr GOM concentrations ≤10^th^ and ≤25^th^ percentiles for each season were excluded from the dataset (GOMqc-10 and GOMqc-25 cases, respectively, in Table 1). The 10^th^ and 25^th^ percentile GOM concentrations at the Dartmouth site are provided in Table 2. Removal of the low GOM measurements in GOMqc-10, GOMqc-25, and GOMqc-50 resulted in higher mean, median, and minimum values than those of the original GOM data (Table 2a and c).

### Analysis

The PCA results from this study were compared to the reference case by examining the similarities and differences in the number of components, component loadings, percent variance explained by each component, and Hg sources or processes inferred. Comparisons of APCS trends by season and trajectory patterns were also performed following the methodology in the reference case, which applied one-way ANOVA to compare the mean APCS between seasons and between trajectory patterns. CWT source locations for RM, GOMqc-10, and GOMqc-25 from this study were compared to the CWT source locations for GOM only and PBM only of the reference cases. The CWT results of the different Hg datasets were correlated with total Hg point source emissions from 2010–2011^21^,^22^ and anthropogenic Hg emissions from the Arctic Monitoring and Assessment Programme (AMAP/UNEP)^23^. The AMAP Hg emissions inventory includes data on point sources as well as non-point sources and Hg speciation^24^,^25^,^26^.

## Results

### Analysis 1: PCA and APCS – Kejimkujik National Park site

#### SpecHg (reference case) vs. RM

**Figure 1:**
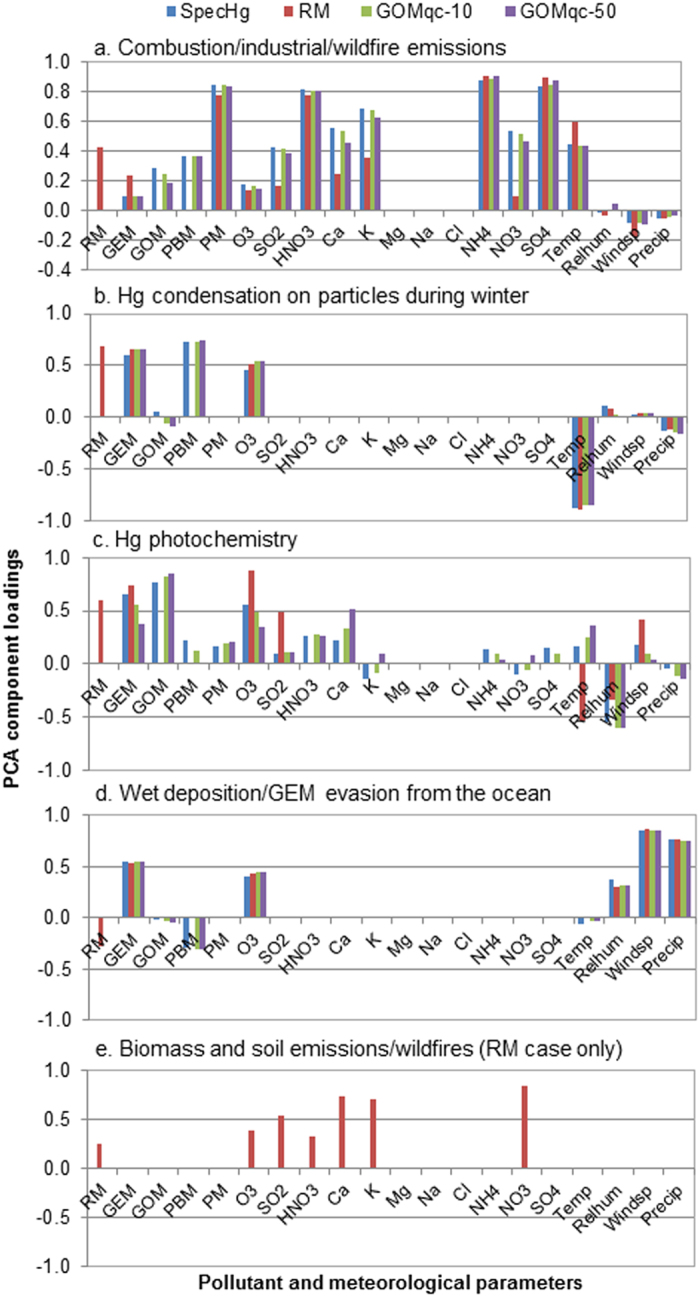
Comparison of the PCA component loadings from this study (RM, GOMqc-10, GOMqc-50 cases) and a previous study (SpecHg case). The percent variance explained by combustion/industrial/wildfire emissions (**a**) was 28.6%, 24.7%, 28.1%, and 27% for the SpecHg, RM, GOMqc-10, and GOMqc-50 cases, respectively. Hg condensation on particles during winter (**b**) explained 24%, 28.3%, 25.4%, and 25.4%. Hg photochemistry (**c**) explained 12.4%, 18%, 12.1%, and 12.0%. Wet deposition/GEM evasion from the ocean (**d**) explained 25.4%, 28.2%, 25%, and 25%. In (**c**), the Hg photochemistry component for the RM case (red bars) was a non-unique component because some parameters in this component also represented Hg condensation on particles during winter. (**e**) is the additional component found in the RM case.

Four components were extracted from PCA of the RM dataset, which is consistent with the number of components from PCA of the SpecHg in a previous study^12^. The three common components in both datasets were combustion/industrial/wildfire emissions, Hg condensation on particles in the winter, and GEM evasion from the ocean. The trend in the component loadings was similar to those of SpecHg (Fig. 1a,b,d). The percent variance of the RM data explained by the components differed from SpecHg by 3–4%, which suggests the use of RM changes the percent variance explained by each component slightly. One of the major discrepancies between the RM and SpecHg results was that one component from the RM dataset can be interpreted as Hg photochemistry as well as Hg condensation on particles during winter (Fig. 1c). In contrast to the SpecHg case, Hg photochemistry and Hg condensation on particles during winter were inferred from separate components. Figure 1e shows an additional component generated from the RM dataset representing biomass and soil emissions (with high loadings on K^+^ and Ca^2+^, explained 16.9% of the variance), which was not obtained from SpecHg. Other minor differences in the component loadings were also observed. For example in the RM dataset, the combustion/industrial/wildfire emissions component had a positive loading on RM and GEM and minor loadings on SO_2_ and NO_3_^−^, while this component had a positive loading on PBM and a near-zero loading on GEM in the SpecHg case. Further details on the differences in the component loadings will be discussed later in the Discussion section.

**Figure 2:**
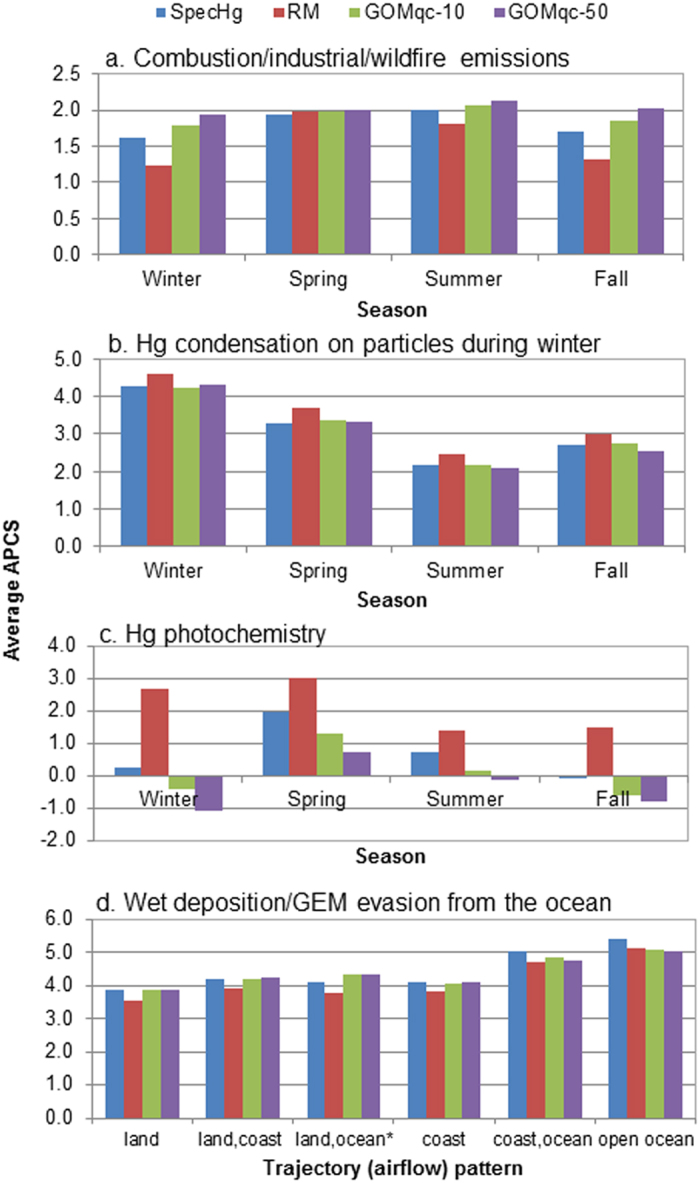
Comparison of average Absolute Principal Component Scores (APCS) analyzed by season and trajectory pattern from this study (RM, GOMqc-10, GOMqc-50 cases) and a previous study (SpecHg case). *Indicates low sample size, N = 3–7 sampling days. In (**c**), the Hg photochemistry component for the RM case (red bars) was a non-unique component because some parameters in this component also represented Hg condensation on particles during winter.

Comparison of the APCS results analyzed by season and trajectory patterns showed strong agreement between RM and SpecHg. Both datasets found that the average APCS for the combustion/industrial/wildfire component was significantly higher in the spring and summer than winter and fall (P < 0.05), which is consistent with the occurrence of wildfires in spring and summer (Fig. 2a). This component was also more strongly associated with coastal airflows traveling over the Canadian and U.S. east coasts than continental and marine airflows (P < 0.05) for both the RM and SpecHg (figure not shown). The combustion sources of Hg were attributed to marine vessels and shipping ports as shown in a previous study^12^. In the Hg condensation component (Fig. 2b), the average APCS for both datasets were consistently higher during winter than in other seasons (P < 0.05), since lower temperatures favor the partitioning of Hg(II) to the particulate phase. In Fig. 2c, the average APCS for the component assigned to Hg photochemistry was greatest in the spring (P < 0.05) for the RM and SpecHg case. The APCS results for both RM and SpecHg found that the GEM evasion from the ocean component was more attributed to marine airflows from the open ocean than continental and coastal airflows (P < 0.05; Fig. 2d).

#### SpecHg (reference case) vs. GOMqc-10 or GOMqc-50

PCA of the GOMqc-10 and GOMqc-50 datasets produced four components that were consistent with the major sources identified from SpecHg. The components represented combustion/industrial/wildfire emissions, Hg condensation on particles in the winter, Hg photochemistry, and GEM evasion from the ocean. The percent variance of the GOMqc-10 and GOMqc-50 datasets explained by each component only differed from SpecHg by 0.3–1.6%. The component profiles among SpecHg, GOMqc-10 and GOMqc-50 shown in Fig. 1 were nearly identical, except for the Hg photochemistry component (further discussion to follow). Similar to the PCA results based on RM data, the APCS trends analyzed by season and trajectory patterns were in agreement with those in the reference case (Fig. 2).

### Analysis 2: CWT model - Dartmouth site

#### GOM or PBM (reference cases) vs. RM

**Figure 3:**
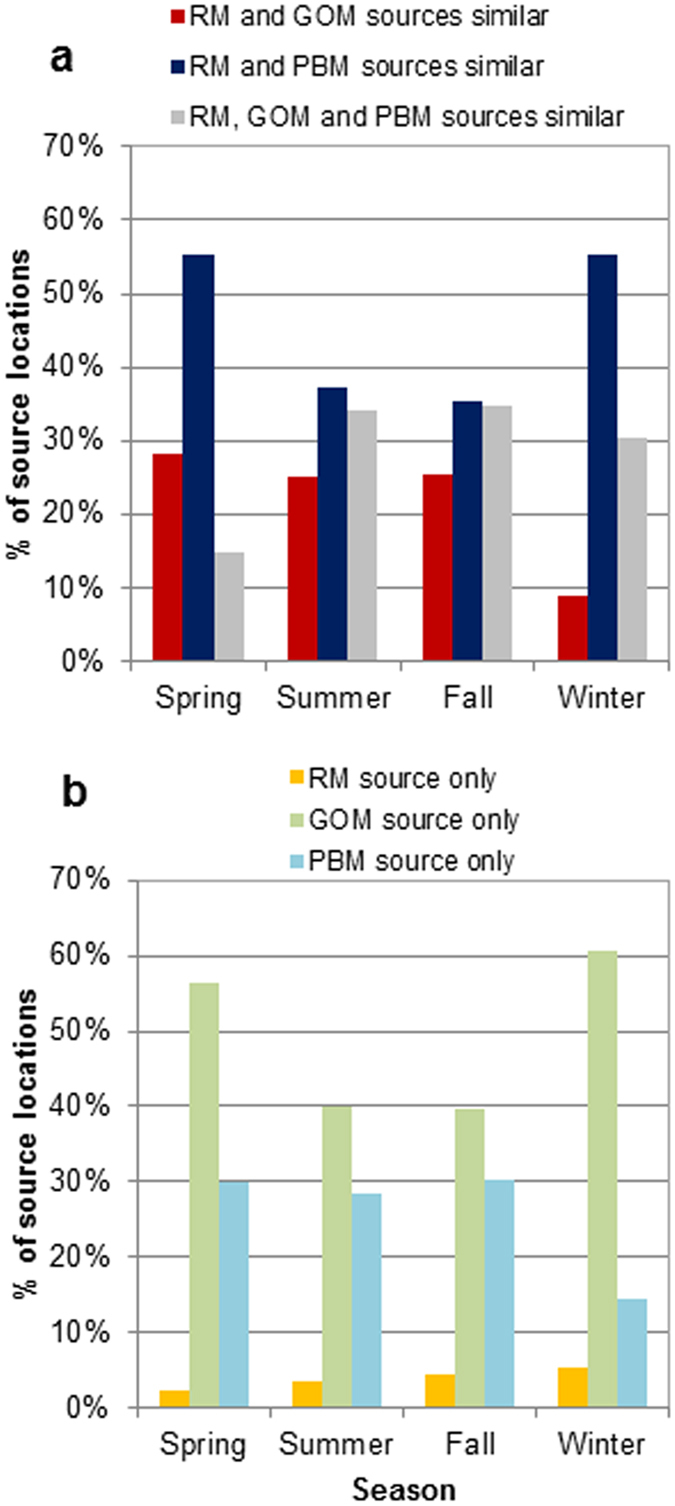
Comparison of CWT modeled source locations from this study (RM case) and previous study (GOM or PBM cases).

**Figure 4:**
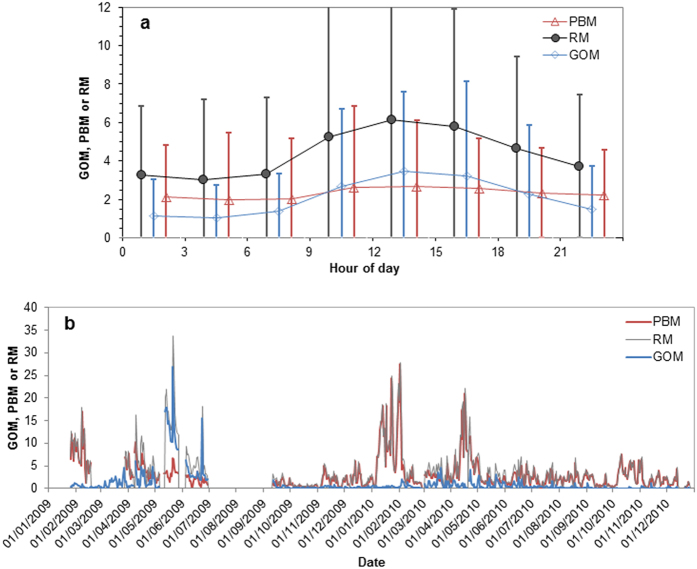
Time-series hour of day average GOM, PBM and RM concentrations (pg m^−3^) at the Dartmouth site (**a**) and daily average GOM, PBM and RM concentrations (pg m^−3^) at the KEJ site (b).Error bars in (**a**) indicate one standard deviation.

In a previous study, the CWT model was used to identify potential source locations contributing to the observed GOM and PBM at the Dartmouth site^13^. Using the same model, the source locations of RM were generated and compared with those of GOM and PBM. The agreement between RM, GOM, and PBM source locations identified in the CWT model ranged from 15–35% depending on the season (Fig. 3a – grey bars). Stronger agreement was found between RM and PBM source locations than those between RM and GOM, since PBM constituted a larger fraction of RM than GOM in most of the seasons except spring (Table 2c and Fig. 4a). Higher degree of agreement between RM and PBM source locations was found in the winter and spring. The RM results identified up to 5% additional source locations that were not identified by either GOM or PBM. In contrast, 40–61% of the GOM source locations were not identified by either PBM or RM (Fig. 3b – green bars). This suggests the importance of the GOM data for identifying additional source areas, particularly in the winter and spring data subsets.

**Figure 5:**
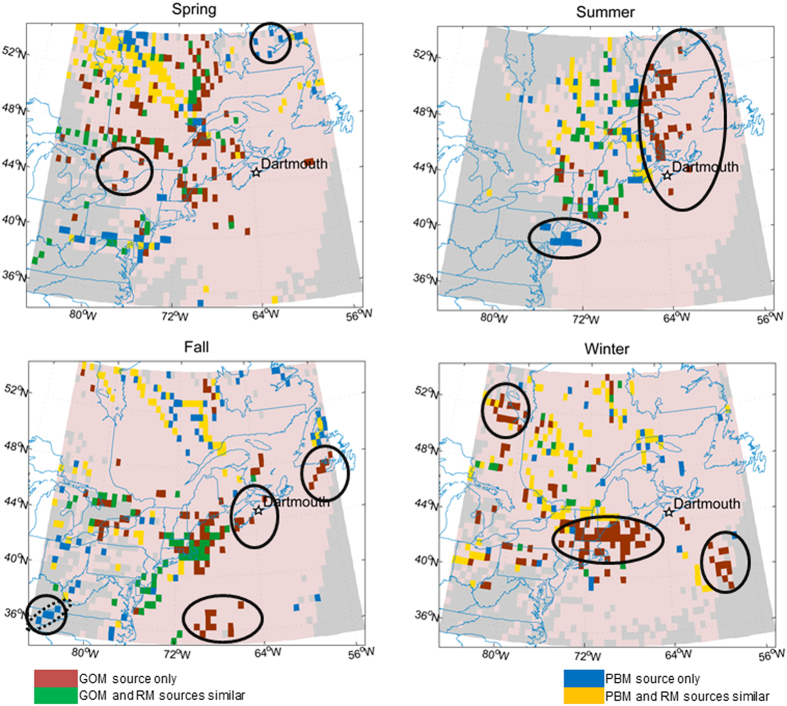
Seasonal CWT modeled source locations based on the results in Fig. 3. Pink areas indicate non-major source locations; grey areas indicate uncertain source locations having <2 trajectory endpoints. Solid circle = isolated source locations of GOM only or PBM only. Dotted circle = Appalachian mountaintop mining. The maps were created using MATLAB.

Source locations identified by the PBM data only were in close proximity to those identified by both RM and PBM data (Fig. 5 blue vs. yellow). Those recognized by the GOM data only were more scattered and isolated (Fig. 5 red vs. green). For example, the GOM data identified additional potential source locations in Newfoundland, Gulf of St. Lawrence, northern and southern Ontario, eastern Quebec, and U.S. northeast that were not identified by RM. PBM data also identified an additional source location near the Appalachian Mountains during fall that was not identified by RM, which may be attributed to mountaintop mining operations.

The Spearman correlation coefficient (r_s_) between total Hg point source emissions and CWT of RM was 0.09 (P = 0.31), which was lower than that of the CWT of PBM for the same site^13^ (r_s_ = 0.27, P = 0.003). However, the low correlation coefficient for the RM data was comparable to that of GEM and GOM. When the CWT results of RM were compared to anthropogenic Hg emissions from AMAP, the correlation coefficient improved slightly (r_s_ = 0.15, P = 0.001). These results suggest that using RM does not significantly improve the accuracy of the CWT model at locating Hg point sources.

#### GOM (reference case) vs. GOMqc-10 or GOMqc-25

Good agreement in the CWT source locations was obtained between the GOM, GOMqc-10 and GOMqc-25 datasets. Depending on the season, 97–99% of the source locations identified was in agreement between the three GOM datasets. Up to 0.5% of the source locations were identified by the GOM data only, while 1–3% of the source locations were identified by the GOMqc-25 data only. These results indicate that removing low GOM measurements in the CWT model did not identify many additional source regions. Most importantly, almost all of the source locations identified by the GOM data in a previous study were also captured by the GOM data excluding low GOM measurements. The reason is likely because high concentrations have more impact on the CWT than low concentrations. Thus, removing the low GOM concentrations had little impact on the CWT results. Selection of a higher cut-off concentration may lead to greater differences in the CWT results; however, that would exclude a large proportion of GOM measurements from this site.

The season with the greatest difference in the CWT results between the three GOM datasets was in the winter. Despite greater differences, the majority of source locations in the winter subset identified by GOMqc-25 only were near (i.e. one grid cell apart) those identified by either of the other two sets of GOM data (full and GOMqc-10). No improvement in the correlation coefficient between the CWT of GOM and total Hg point source emissions was found when the quality-controlled GOM data was evaluated (r_s_ = 0.13–0.14, P = 0.11–0.14). Comparison of the CWT for the various GOM datasets with AMAP Hg emissions also resulted in weak correlation coefficients (r_s_ < 0.02, P > 0.5).

## Discussion

Correlation analysis provided more insight on the comparison of PCA results between RM and SpecHg. In all three common components shown in Fig. 1a,b,d, the loadings of RM were similar to those of PBM in the SpecHg case, indicating RM’s relationship with other parameters is more similar with those of PBM than of GOM, as supported by correlations in Table 3. The similarity with PCA loadings can be explained by the large PBM percentage of RM as shown in Fig. 4b (average 80%). PBM accounted for a large majority of RM and the trends of RM and PBM were similar in most dates. The low correlation coefficient between RM and PBM was likely caused by a few low PBM episodes in May and June of 2009 (Fig. 4b). Some differences in the component loadings for the combustion/industrial/wildfire emissions component was also reflected in the correlation results. RM was more strongly correlated with GEM than PBM or GOM (Table 3), which is consistent with the greater component loading on GEM when RM was used (Fig. 1a).

Furthermore, PCA results were more sensitive to the use of RM. In PCA with RM, one component could be assigned to both Hg condensation on particles during winter and Hg photochemistry (Fig. 1c). Hg condensation on particles was inferred from positive loadings on GEM, RM and negative loadings on temperature, similar to that of the SpecHg case except for the presence of PBM instead of RM. Hg photochemistry in this component was inferred by positive leadings of RM, O_3_ and a negative loading of relative humidity, similar to the SpecHg case except for the presence of GOM instead of RM. This non-unique component was due the shared correlations between RM and GOM and between RM and PBM. Overall both correlation coefficients are fairly strong (Table 3). However, the RM-PBM correlation was especially higher during the cold seasons (from December to end of April, r = 0.96, P < 0.0001), which is also when there was an inverse correlation between PBM and temperature. This likely resulted in the inverse correlation between RM and temperature, which characterizes gaseous Hg condensation under lower temperatures. At the same time, the pollutants/met parameters (ozone, relative humidity) that were correlated with GOM, which were used to characterize Hg photochemistry, were also correlated with RM because of the strong RM-GOM correlation. APCS results for RM in Fig. 2c also indicated that this non-unique component dominated in the colder seasons (i.e. winter/spring), suggesting both Hg processes may be occurring simultaneously. Temperatures in Atlantic Canada are still cold in the spring, which is conducive to particle partitioning of gaseous Hg. Buildup of pollutants (e.g. ozone precursors) during winter will undergo photochemical reactions to produce ozone as the solar radiation intensity begins to rise in the spring. This is consistent with the higher average ozone during spring and winter than summer and fall in the coastal eastern Canada region^27^. Additional high loadings were found for SO_2_ and wind speed (Fig. 1c); the former is supported by strong correlations between RM and SO_2_ (Table 3). There was an additional component extracted from the RM dataset attributed to biomass and soil emissions due to strong loadings on K^+^ and Ca^2+^ (Fig. 1e). Correlation analysis indicated stronger correlations between K^+^ and Ca^2+^ with RM than with PBM (Table 3), which may explain the presence of this additional component with strong loadings on RM, K^+^ and Ca^2+^ for the RM dataset. Based on the pollutant markers, it may represent both biomass and soil emissions and forest fires.

Overall, excluding low GOM data had minor effects on PCA and APCS results. The major components were easily assigned to the same Hg sources and processes identified in a previous study using the full dataset; however, some differences were found in the component loadings. Excluding low GOM measurements (GOMqc-10 and GOMqc-50 cases) from the dataset resulted in higher loadings on GOM, Ca^2+^, and temperature and weaker loadings on GEM and O_3_ in the Hg photochemistry component (Fig. 1c). This is likely because correlation coefficients between daily average GOMqc-10 or GOMqc-50 and temperature were also slightly higher than that between GOM and temperature (Table 3). Because this component was interpreted as Hg photochemistry, a strong anti-correlation between GOM and GEM should be expected^28^,^29^; instead a weaker positive correlation between GOMqc-10 or GOMqc-50 and GEM was observed. These findings suggest removing low GOM measurements is conducive to extracting a Hg photochemistry component. This is likely because the higher GOM measurements retained in the dataset occurred during the daytime period when higher solar radiation intensity favors GEM oxidation. The effect on correlation and PCA results may be more pronounced if PCA were applied to the 3-hr average dataset, instead of daily averages. Although the two approaches (using RM and removing low GOM measurements) may improve the data quality and reproduce the Hg sources and processes from a previous study, it is still uncertain whether the PCA results are more accurate because the sources inferred from PCA cannot be verified independently.

The correlation between CWT of RM and anthropogenic Hg emissions from AMAP improved compared to the CWT of GOM in the reference case. But compared to the CWT results of PBM in the reference case, the correlation between CWT results of RM and Hg emissions was lower. This was expected because GOM and PBM concentrations were summed but there was no change to the trajectory residence time of each grid cell. In cases where low GOM concentrations were removed (GOMqc-10 and GOMqc-25), the weak correlations observed between CWT results and Hg emissions were nearly the same as that of the reference case. A lack of difference in these results was likely due to the overall low GOM concentrations at the Dartmouth site. Removing low GOM concentrations had little impact on the CWT results because only higher concentrations would have greater impact on the CWT. Another reason is that the sources of GOM at the Dartmouth site may not be attributed directly to anthropogenic sources because atmospheric Hg can convert between the different forms in the atmosphere and undergo deposition. Furthermore, uncertainties in the back trajectory distances caused by starting position of the trajectory, turbulent airflows, modeled vertical motion and wind fields, etc. will impact the CWT results^13^,^30^,^31^.

## Conclusions

The effects of the uncertainties in GOM measurements on Hg receptor model results were assessed. One method of reducing uncertainties in GOM involves combining GOM and PBM data into a single parameter, RM. The second method excludes lower percentile measurements of GOM. The study found PCA and APCS model results using either method were in agreement with results from previous studies using the original dataset. The percentage variance explained by each of the major components in a previous PCA study differed slightly (0.3–1.6%) compared to the dataset excluding low GOM measurements and differed by 3–4% compared to the RM dataset. The RM dataset also extracted a non-unique component and an additional component that was not present when PCA was performed with GOM and PBM as separate parameters.

In the CWT model, the location of the source regions of GOM in a previous study differed slightly (up to 0.5%) compared to GOM data excluding low measurements, but differed significantly (40–61%) compared to RM, depending on the season analyzed. Comparison of the CWT results with anthropogenic Hg emissions did not greatly improve the accuracy of the CWT model results when quality-controlled GOM data or when RM data were used. Future studies could evaluate the CWT results with a more comprehensive bottom-up mercury emissions inventory.

A potential advantage of using GOM data over RM in the CWT model is the identification of additional source locations, whereas PBM data tend to identify source locations in proximity to those of PBM and RM. Therefore, while RM is considered a more accurate parameter from a measurement point-of-view, receptor modeling using speciated Hg (GEM, GOM, and PBM) has continued to improve our knowledge of Hg sources, transformation, and deposition. This trade-off will be further investigated in a future study by comparing receptor modeling results between GEM and speciated Hg datasets, given the relatively more accurate GEM measurements. Future studies could apply both data treatment methods at other sites to investigate the validity of conclusions drawn in this study. Moreover, the use of 2–3 hr averaged Hg data instead of daily means in this study may provide more insight about the effects of using RM on the PCA model’s ability to delineate components with strong diurnal variability such as photochemistry. In the meantime, the approach of removing low GOM measurements prior to receptor modeling may reduce uncertainties in the dataset and has minimal effect on receptor modeling results.

## Additional Information

**How to cite this article**: Cheng, I. *et al.* Impact of Measurement Uncertainties on Receptor Modeling of Speciated Atmospheric Mercury. *Sci. Rep.* **6**, 20676; doi: 10.1038/srep20676 (2016).

## Figures and Tables

**Table 1:** Summary of receptor modeling scenarios.

ANALYSIS 1	Site	Receptor models	Dataset
Data and model description	Kejimkujik National Park, Nova Scotia, Canada (KEJ site)	principal components analysis (PCA) and absolute principal components scores (APCS)	Daily average dataset from 2009–2010
Modeling scenarios	Speciated atmospheric Hg (SpecHg^a^) - reference case in Cheng *et al.*^12^		
	Reactive Hg (RM)		
	Excluding GOM concentrations ≤10th percentile (GOMqc-10)		
	Excluding GOM concentrations ≤50th percentile (GOMqc-50)		
**ANALYSIS 2**			
Data and model description	Dartmouth, Nova Scotia, Canada	concentration-weighted trajectory model (CWT)	3-hr dataset from 2010–2011
Modeling scenarios	GOM or PBM - reference case in Cheng *et al.*^13^		
	Reactive Hg (RM)		
	Excluding GOM concentrations ≤10th percentile in each season (GOMqc-10)		
	Excluding GOM concentrations ≤25th percentile in each season (GOMqc-25)		

^a^SpecHg case: GOM and PBM were separate parameters in the dataset used in PCA.

**Table 2:** Summary Hg concentration statistics (pg m^−3^) in the different datasets.

(a) KEJ site	Daily average in 2009				Daily average in 2010			
*Original data in reference case*	Mean	Median	Min	Max	Mean	Median	Min	Max
GOM	1.8	0.4	0	27.0	0.4	0.2	0	4.7
PBM	2.8	2.1	0	17.1	3.4	2.2	0.1	27.5
*Modified data*								
RM	4.7	3.0	0	33.7	3.8	2.6	0.2	27.9
GOMqc-10	2.3	0.7	0.1	27.0	0.8	0.5	0.1	4.7
GOMqc-50	2.7	0.9	0.3	27.0	1.1	0.7	0.3	4.7
**(b) Dartmouth site**	# of 3 hr measurements							
*Original data in reference case*	Winter	Spring	Summer	Fall				
GOM	1236	1236	1385	1319				
PBM	1210	1190	1211	1319				
*Modified data*								
RM	1210	1190	1211	1319				
GOMqc-10	1112	1112	1246	1186				
GOMqc-25	927	927	1037	988				
**(c) Dartmouth site**	Median of 3 hr data				Median % of RM			
*Original data in reference case*	Winter	Spring	Summer	Fall	Winter	Spring	Summer	Fall
GOM	1.0	1.7	1.0	0.8	36%	53%	44%	39%
PBM	1.8	2.0	1.4	1.4	64%	47%	56%	61%
10th percentile GOM	0.25	0.33	0	0				
25th percentile GOM	0.55	0.68	0.33	0.33				
*Modified data*								
RM	3.0	4.0	2.7	2.6				
GOMqc-10	1.2	2.0	1.3	1.0				
GOMqc-25	1.4	2.6	1.4	1.2				

**Table 3:** Pearson correlation coefficients (r) between daily average Hg and other pollutant and meteorological parameters at the KEJ site.

	GOM	PBM	RM	GOMqc-10	GOMqc-50
GEM	0.38	0.39	0.50	0.35	0.33
GOM					
PBM	0.17			*0.10*	*0.04*
RM	0.86	0.65			
O_3_	0.24	0.59	0.54	0.22	0.18
PM_2.5_	0.29	0.48	0.49	0.28	0.27
SO_2_	0.23	0.64	0.52	0.22	0.19
HNO_3_	0.41	0.43	0.56	0.40	0.39
Ca^2+^	0.37	0.27	0.43	0.37	0.39
K^+^	0.18	0.13	0.20	0.16	0.17
NH_4_^+^	0.24	0.51	0.47	0.22	0.20
NO_3_^−^	0.14	0.49	0.38	*0.11*	*0.09*
SO_4_^2−^	0.22	0.50	0.45	0.19	0.17
Temp	0.37	−0.44	*0.05*	0.40	0.41
Relhum	−0.36	−0.38	−0.49	−0.33	−0.30
Windsp	*0.00*	*0.01*	*0.02*	−0*.01*	−0*.03*
Precip	*−0.08*	*−0.12*	*−0.12*	*−0.08*	*−0.07*
